# Analysis of the UK Government’s 10-Year Drugs Strategy—a resource for practitioners and policymakers

**DOI:** 10.1093/pubmed/fdac114

**Published:** 2022-10-29

**Authors:** Adam Holland, Alex Stevens, Magdalena Harris, Dan Lewer, Harry Sumnall, Daniel Stewart, Eilish Gilvarry, Alice Wiseman, Joshua Howkins, Jim McManus, Gillian W Shorter, James Nicholls, Jenny Scott, Kyla Thomas, Leila Reid, Edward Day, Jason Horsley, Fiona Measham, Maggie Rae, Kevin Fenton, Matthew Hickman

**Affiliations:** Bristol Medical School, University of Bristol, Bristol, BS8 2BN, UK; School of Social Policy, Sociology and Social Research, University of Kent, Canterbury, CT2 7NZ; Department of Public Health, Environments and Society, London School of Hygiene & Tropical Medicine, London, WC1E 7HT, UK; Public Health Specialty Registrar, Department of Epidemiology and Public Health, University College London, London, WC1E 6BT, UK; Public Health Institute, Liverpool John Moores University, Liverpool, L3 5UX, UK; Bristol Medical School, University of Bristol, Bristol, BS8 2BN, UK; Population Health Sciences Institute, Faculty of Medical Sciences, Newcastle University, Newcastle, NE1 7RU, UK; Association of Directors of Public Health, London, EC4Y 0HA, UK; Bristol Medical School, University of Bristol, Bristol, BS8 2BN, UK; Association of Directors of Public Health, London, EC4Y 0HA, UK; School of Psychology, Queen’s University Belfast, Belfast, BT7 1NN; Faculty of Health Sciences and Sport, University of Stirling, Stirling, FK9 4LA, UK; Department of Pharmacy & Pharmacology, University of Bath, Bath, BA2 7AY; Bristol Medical School, University of Bristol, Bristol, BS8 2BN, UK; Hepatitis C Trust, London, SE1 3YD, UK; Institute of Mental Health, University of Birmingham, Birmingham, B15 2TT; National Institute for Health Research Evaluation Trials and Studies Coordinating Centre, University of Southampton, Southampton, SO17 1BJ, UK; Department of Sociology, Social Policy and Criminology, University of Liverpool, Liverpool, L69 3BX; Epidemiological and Public Health Section, Royal Society of Medicine, London, W1G 0AE, UK; Faculty of Public Health, London, NW1 4LB, UK; Bristol Medical School, University of Bristol, Bristol, BS8 2BN, UK

**Keywords:** addiction, Government and Law, public health

## Abstract

In 2021, during a drug-related death crisis in the UK, the Government published its ten-year drugs strategy. This article, written in collaboration with the Faculty of Public Health and the Association of Directors of Public Health, assesses whether this Strategy is evidence-based and consistent with international calls to promote public health approaches to drugs, which put ‘people, health and human rights at the centre’. Elements of the Strategy are welcome, including the promise of significant funding for drug treatment services, the effects of which will depend on how it is utilized by services and local commissioners and whether it is sustained. However, unevidenced and harmful measures to deter drug use by means of punishment continue to be promoted, which will have deleterious impacts on people who use drugs. An effective public health approach to drugs should tackle population-level risk factors, which may predispose to harmful patterns of drug use, including adverse childhood experiences and socioeconomic deprivation, and institute evidence-based measures to mitigate drug-related harm. This would likely be more effective, and just, than the continuation of policies rooted in enforcement. A more dramatic re-orientation of UK drug policy than that offered by the Strategy is overdue.

## Introduction

In 2021, the UK Government published its 10-year drugs strategy, *From Harm to Hope*^1^ (hereafter referred to as ‘the Strategy’) following Dame Black’s Independent Review of Drugs.^2^ This is during a period of escalating drug-related deaths in the UK^3–5^ surpassing the rates of many countries.^6^ The following analysis, undertaken with the Faculty of Public Health and Association of Directors of Public Health, assesses whether the Strategy is evidence-based and consistent with the call from the highest coordination forum of the United Nations (UN) to ensure drug strategies promote public health and human rights.^7^ The Strategy is structured under three strategic priorities: to ‘Break drug supply chains’, ‘Deliver a world-class treatment and recovery system’, and ‘Achieve a generational shift in demand for drugs’. This article discusses drug-related harm in the UK, the Strategy’s three pillars, and highlights missing elements of policy.

### Drug-related harm in the UK

‘Drug-related harm’ encompasses the negative health and social impacts associated with illicit drug use, and drug market involvement. As acknowledged by the Strategy, and the Black Review,^2^ which preceded it, current approaches have not effectively reduced many of these harms. Various health and social issues including socioeconomic deprivation, mental and physical health problems, stigma, trauma and homelessness may both predispose to and be exacerbated by drug dependence.^8–10^

Amongst the health harms related to drug use, drug-related deaths provide the most obvious metric. Between 2010 and 2019, age standardized drug-related mortality rates increased in Scotland by 171% (from 90 to 244 per million)^3^; Northern Ireland by 149% (from 35 to 87 per million)^4^; and England and Wales by 61% (from 31 to 49 per million).^5^ There are likely multiple reasons for these increases.^2^^,^^11^ A common argument is that deaths increased because people with drug dependencies are older, with comorbidities increasing overdose risk. Two recent studies, however, demonstrated ageing alone does not explain the increase.^12^^,^^13^ Other potential contributory factors include: (i) increasing polydrug use, with the risk of opioid overdose increasing with concomitant benzodiazepine, gabapentinoid, and alcohol use^14–17^; (ii) increasing homelessness and incarceration, which are associated with mortality risk, and human immunodeficiency virus (HIV) and hepatitis C (HCV) transmission^9^^,^^10^^,^^18^; (iii) changing patterns of socioeconomic deprivation, which is strongly associated with drug-related harm^19–21^ and (iv) cuts to services that protect against all-cause and drug-related mortality.^2^^,^^21^^,^^22^

The Strategy makes some unsupported assumptions about the relationship between drugs and social problems. It suggests that drugs ‘blight’ neighbourhoods, stopping them from reaching their potential, implying drugs cause socioeconomic deprivation as opposed to the latter creating conditions in which drug markets flourish. Socioeconomic deprivation and adverse childhood experiences are inter-related^23^ with both associated with harmful patterns of drug use,^19^^,^^20^^,^^24^ Furthermore, disinvestment in health and social services in socioeconomically deprived areas since 2010 may have contributed to increasing harm.^22^

Drug-related harms to third parties include acquisitive crime and drug-related violence. However, in some instances the Strategy exaggerates the causative relationship between drugs and crime. For example, it states drugs ‘contribute’ to almost half of all homicides, seemingly implying causation. In 2020, 48% of homicides were in some way related to drugs—in most cases, the victim or perpetrator was known to use or deal drugs, sometimes recently.^25^ In a small proportion of cases, motives were related to obtaining drugs or drug proceeds,^25^ but for the most part, it is not clear that drugs caused the homicides, and in no cases is it clear stricter drug controls would have prevented them.

### Breaking drug supply chains

The first pillar of the strategy aims to: reduce drug availability by targeting supply chains, including international, wholesale and retail providers, with a particular focus on ‘county lines dealing’ (when drugs are transported from cities to other areas, and sold using a mobile phone ‘line’).

There is some evidence that limiting the supply of a drug increases its purity adjusted price,^26^ which can reduce demand for that drug,^27^ thereby reducing hospital attendances and overdoses related to its use.^28^ There are, however, three issues with enforcement-led efforts to reduce drug supply.

First, there is limited evidence of their effectiveness. The Government has highlighted there is a lack of relevant evaluative research,^29^ and available evidence does not suggest that arresting dealers or seizing drugs has a long-term impact on supply.^30^ Internationally, there have been some isolated reductions in drug supply, for example, after global market disruption interrupted heroin supply in Australia in 2000^31^ and Western Europe in 2010^32^; and controls on precursor chemicals in the USA in 1989 and 2006 impacted cocaine availability.^33^ These reductions were, however, temporary, and it is not clear what caused them when other efforts have not had the same impact. Despite recent seizures, global production and purity of drugs continues to increase^34^ and the UK has amongst the cheapest heroin and cocaine in Europe.^35^

Second, there is limited understanding of how restricting the supply of certain drugs affects the supply of, demand for, and harm related to other drugs. For example, during ‘droughts’ of specific drugs, people may use adulterated drugs, alternative drugs, or resort to polydrug use.^36–39^

Third, enforcement may have unintended consequences on the drug market and people who use drugs, leading to increased harm. Focussing on the most violent and exploitative forms of supply, such as those associated with county lines dealing^40^ may shape the market to adopt less harmful practices.^41^^,^^42^ However, as the Black Review highlighted,^2^ arresting suppliers can create conditions that favour competition, promoting innovation and violence.^41–51^

### Delivering a world-class treatment and recovery system

The second pillar of the Strategy aims to: rebuild treatment services following significant disinvestment; promote integration of drug treatment, health and criminal justice services; and improve employment and accommodation opportunities.

Additional drug treatment funding promised by the Strategy is welcome; however, this follows years of sustained disinvestment,^2^ associated with reductions in numbers of people in treatment,^52^ and an increase in the proportion of people using opioids and crack cocaine not engaged with services.^53^ Furthermore, drugs workers have experienced increasing caseloads and greater administrative responsibilities, sometimes limiting their capacity to provide psychosocial interventions.^54^

The Strategy suggests ‘recovery from drug addiction’ is a key aspect of its approach. As the UK Government Recovery Champion highlights, recovery and harm reduction should not be considered as opposing approaches, and the full range of evidence-based interventions should be provided.^55^ Opioid agonist therapy (OAT—treatment of opioid dependence with methadone or buprenorphine) reduces the risks of all-cause mortality, overdose, suicide, self-harm, HIV and HCV, improves quality of life^9^^,^^56–61^ and duration of OAT improves survival.^62–65^ Whilst modelling demonstrates comprehensive OAT and harm reduction programmes reduce drug-related mortality,^66–68^ this is dependent on retention in treatment, which should be a key indicator. Focusing on treatment completion may incentivise premature OAT cessation, limiting treatment benefits and the impact of additional funding.

Increased funding and targeted commissioning could allow the introduction of innovative interventions, including drug checking and diamorphine-assisted treatment—neither of which the Strategy mentions. No intervention alone will avert the drug-related death crisis, but in combination with wider treatment systems, these evidence-based interventions could have beneficial impacts on patterns of harm.^69–72^ Local areas may need additional funding and technical support to commission diamorphine-assisted treatment, which is more expensive than oral OAT.^73^ These costs, however, are compensated by greater savings to wider services, including related to reductions in acquisitive crime.^73^ Drug checking, on the other hand, is expanding, as the UK’s first regular Home Office licensed, local authority funded drug checking service launches in Bristol.^74^

Despite their relevance, the Strategy does not mention HCV and HIV prevention. An estimated 89% of people infected with HCV in the UK have injected drugs^75^ and a recent outbreak of HIV occurred amongst people who inject drugs in Glasgow.^76^ The UK is a leader in providing HCV treatment for people who inject drugs, with clear reductions in chronic infections and liver-related deaths.^12^^,^^77–80^ However, achieving the World Health Organization (WHO) target of ‘eliminating HCV as a public health problem’^75^ will depend on preventing reinfection, with HCV infection a critical indicator for assessing the success of drug treatment and harm reduction systems.^81^

People with drug dependencies often have co-occurring health problems. People in drug treatment are getting older, and more deaths are caused by long-term conditions than overdoses.^12^^,^^82^ Office for Health Improvement and Disparities data suggest 63% of people starting drug treatment have a mental health need^53^ and people with substance dependence are at greater risk of suicide.^83^ A recent study demonstrated that one in fourteen opioid-related deaths in England occur amongst people recently discharged from hospital,^84^ highlighting the need to improve integration between healthcare and drug treatment services. Drug services will need to recruit more clinically trained staff to identify and manage co-occurring health issues, which will be challenging as the workforce has been depleted by disinvestment. Furthermore, hospital care for people with drug dependence requires improvement. Stigmatizing attitudes towards people who use drugs and fear of opioid withdrawal are key barriers to healthcare access,^85–87^ underpinned by hospital policies that create significant procedural barriers to providing OAT.^88^

Drug-related harm remains a key issue in prison, with overdose risk substantially elevated in the month following release^89–91^ and incarceration a risk factor for HIV and HCV.^18^ Prison OAT reduces mortality and drug use in prison and critically also mortality following release.^92^^,^^93^ The Strategy’s proposed zero-tolerance approach to drugs is inconsistent with the Inspectorate of Prisons acknowledgement of the importance of harm reduction strategies in prisons.^94^ Proposed alternatives to prison OAT, including detoxification, are experimental, and it is necessary to demonstrate they do not increase drug-related deaths (during and after incarceration) compared to OAT. Evaluations of previous Drug Recovery Wings, which utilized abstinence and harm reduction-based approaches, highlighted potential benefits but identified challenges, particularly related to limited support on release.^95^ The Strategy recognizes the need for improved inter-agency coordination during and following incarceration, however recommendations from the Advisory Council on the Misuse of Drugs (ACMD) to improve custody-community transitions have not been realized.^96^

### Achieving a generational shift in the demand for drugs

The third pillar of the Strategy aims to: reduce demand for drugs by applying ‘tougher and more meaningful consequences’ to deter use, delivering education programmes in schools and supporting at risk families.

The assumption that the threat of punishment will reduce demand is not supported by evidence, with no clear relationship between the stringency of drug laws and drug use prevalence.^97–101^ The Home Office previously concluded ‘levels of drug use are influenced by factors more complex and nuanced than legislation and enforcement alone’.^102^ These may include socioeconomic deprivation^19^ and adverse childhood experiences^24^; factors that may be exacerbated by the health and social harms associated with contact with the criminal justice system.^103^ Additionally, the stigma associated with punitive policies may deter people with drug dependence from seeking support.^104^

The Strategy’s proposed ‘tough consequences out of court disposal schemes’ provide an opportunity to divert people from the criminal justice system. Available evidence tentatively suggests diversion schemes reduce re-offending more effectively and cost-effectively than criminal sanctions.^105–108^ However, there is limited research evaluating their impacts on drug-related harms^109^ and existing diversion schemes vary in approach and ethos. Whilst diversion schemes may mitigate some of the harms associated with criminal sanctions, most are still designed to negatively impact people who use drugs, which may exacerbate the issues predisposing to harmful use.

The Government’s subsequent White Paper, *SWIFT, CERTAIN, TOUGH* (in consultation), proposes escalating consequences for drug possession including: mandatory drugs awareness courses, random drug testing (and expansion of drugs tested for on arrest), passport and driving licence confiscation, wearable drug monitors and exclusion orders prohibiting attendance of particular venues.^110^ These proposals raise significant concerns. Mandatory drugs awareness courses will require payment, with non-attendance and non-payment punished with fines or criminal charges, placing an inequitable burden on the socioeconomically deprived, who are the most likely to be caught. Passport and driving license confiscations may affect employment prospects and will disproportionately impact the rights of people who use drugs. The intention to ensure ‘more people face consequences of their use’ with expanded drug testing is likely to ‘widen the net’, with more people receiving punishments that may escalate to criminal sanctions with questionable justification. Furthermore, the Strategy implies people could be coerced into drug treatment, contravening human rights and medical ethics norms,^111^ with limited evidence that coerced treatment reduces future drug use.^111^^,^^112^

It remains to be seen how proposed schemes will contribute to the stigma faced by people who use drugs, and whether they will reproduce the ethnic and socioeconomic disparities apparent in current enforcement. People who are black are nearly nine times more likely to be stopped and searched for drugs than people who are white and are more likely to be arrested, prosecuted, and sentenced to immediate custody.^113^ Whilst the Strategy recognizes the problem of disproportionate policing, plans to expand punishments that inequitably impact the socioeconomically deprived do not align with efforts to reduce inequalities and ‘level up’ communities.^114^

### What’s missing?

The Strategy states it is taking a new approach; however, most elements are a continuation of former approaches proposed in the context of existing legislation, rather than allowing for legislative reform to decriminalize the possession of drugs and facilitate innovative interventions.

The Strategy suggests that decriminalization risks increasing drug use; however, this is not supported by evidence.^97–101^ Whilst criminalization has no clear benefits, it causes significant harm to people who use drugs.^115^ Since the Misuse of Drugs Act 1971 was introduced, more than three million criminal records have been generated for drugs offences.^116^ In 2017, 60% of prosecutions for drug offences in England and Wales were for possession rather than supply, including 36% for the possession of cannabis.^113^ In the UK, decriminalization has been recommended by bodies including the 2019 Health and Social Care Committee on Drug Policy^117^; the Royal College of Physicians^118^; the Royal Society of Public Health and the Faculty of Public Health.^119^ Internationally, over 30 countries have some degree of decriminalization,^101^ and it has been recommended by the highest coordination forum of the UN, comprising the Executive Heads of organizations including the WHO and the UN Office for Drugs and Crime.^7^

The Government has resisted the introduction of overdose prevention centres,^120^ despite promising evidence they could reduce drug-related deaths and engage the most marginalized with services.^70^^,^^121^ The introduction of pilot sites has been recommended by numerous health, academic and third sector organizations,^122^^,^^123^ the ACMD,^21^ the 2019 Health and Social Care Committee on Drugs Policy^117^ and the Scottish Drug Deaths Taskforce.^124^ Although overdose prevention centres may be provided in the UK with agreement from local agencies,^125^ legislative change would facilitate pilots, allowing evaluations of their effectiveness and cost-effectiveness.^120^ Currently, legislation also creates barriers to providing smoking paraphernalia to engage people who use crack cocaine with services,^126^ as is the case in other countries.^127^

There was no opportunity for public consultation in the Strategy’s development. For other health and social policies, research and commissioning, the views of the public are included as a matter of priority.^128^^,^^129^ Generally, the views of people who use drugs, who entreat that there should be ‘nothing about us without us’,^130^ have not been adequately considered when developing drugs strategies.^131^ Communities of people who use drugs, and UN agencies, have highlighted human rights implications, including the right to non-discrimination, should be a primary consideration in developing drug strategies.^7^^,^^132^^,^^133^ The Strategy does not mention human rights, and punitive policies and restrictions on access to harm reduction programmes are often at odds with human rights norms.^115^^,^^134^

Stigma related to drug use, including that propagated by the language used to describe people who use drugs,^135^ creates barriers to seeking support.^104^ The Strategy identifies the need to reduce stigma. However, the Government has also suggested that stigma is a valued means to deter drug use initiation.^136^ Elements of the Strategy could be seen as promoting stigma, for example referring to acquisitive crime in terms of’[t]he innocent families whose homes are broken into by addicts seeking to feed their habits’.^1^ Independent anti-stigma campaigns have been launched,^137^^,^^138^ but the evidence for their effectiveness is limited,^139^ as sources of stigma are complex,^140^ and efforts would need to translate into policy and practice to have meaningful impact.

## Conclusion

There are significant inconsistencies between the Strategy and the call from the highest coordination forum of the UN to promote public health approaches to drugs, putting ‘people, health and human rights at the centre’.^7^ A public health approach should tackle upstream factors predisposing to harmful drug use alongside many other health and social disadvantages. Whilst promised investment in drug treatment is welcome and likely to be beneficial, this alone will not solve the drug-related death crisis. Realizing the potential benefits of additional funding and achieving the ambition to develop a ‘world class treatment and recovery system’ will depend on addressing fundamental flaws in the Strategy’s approach. Furthermore, an effective public health strategy should reflect best evidence. Whilst the Strategy states evidence is ‘at the heart’ of its approach, this is not always the case as it continues to promote un-evidenced and harmful measures to deter drug use with punishment.

We believe a public health approach to drugs would be more effective than policies rooted in criminalization and enforcement. Framing drug use as something deserving of punishment promotes stigmatizing attitudes, which pose a barrier to accessing support and approaches that do not adequately consider the views and human rights of people who use drugs. For more than fifty years, this has failed to effect improvements and a more dramatic re-orientation of the UK response to drugs is overdue.

## Data Availability

No new data were generated or analyzed in support of this research.
